# Molecular strategies for the utilisation of human milk oligosaccharides by infant gut-associated bacteria

**DOI:** 10.1093/femsre/fuad056

**Published:** 2023-10-04

**Authors:** Leonie Jane Kiely, Kizkitza Busca, Jonathan A Lane, Douwe van Sinderen, Rita M Hickey

**Affiliations:** Teagasc Food Research Centre, Moorepark, Fermoy, Cork P61C996, Ireland; Health and Happiness Group, H&H Research, National Food Innovation Hub, Teagasc Moorepark, Fermoy, Co. Cork P61K202, Ireland; APC Microbiome Ireland, Biosciences Institute, University College Cork, Cork T12 YT20, Ireland; School of Microbiology, University College Cork, Cork T12 YN60, Ireland; Health and Happiness Group, H&H Research, National Food Innovation Hub, Teagasc Moorepark, Fermoy, Co. Cork P61K202, Ireland; Health and Happiness Group, H&H Research, National Food Innovation Hub, Teagasc Moorepark, Fermoy, Co. Cork P61K202, Ireland; APC Microbiome Ireland, Biosciences Institute, University College Cork, Cork T12 YT20, Ireland; School of Microbiology, University College Cork, Cork T12 YN60, Ireland; Teagasc Food Research Centre, Moorepark, Fermoy, Cork P61C996, Ireland; APC Microbiome Ireland, Biosciences Institute, University College Cork, Cork T12 YT20, Ireland

**Keywords:** human milk oligosaccharides, prebiotics, infant gut microbiota, probiotics, Bifidobacteria, *Lactobacillus*

## Abstract

A number of bacterial species are found in high abundance in the faeces of healthy breast-fed infants, an occurrence that is understood to be, at least in part, due to the ability of these bacteria to metabolize human milk oligosaccharides (HMOs). HMOs are the third most abundant component of human milk after lactose and lipids, and represent complex sugars which possess unique structural diversity and are resistant to infant gastrointestinal digestion. Thus, these sugars reach the infant distal intestine intact, thereby serving as a fermentable substrate for specific intestinal microbes, including Firmicutes, Proteobacteria, and especially infant-associated *Bifidobacterium* spp. which help to shape the infant gut microbiome. Bacteria utilising HMOs are equipped with genes associated with their degradation and a number of carbohydrate-active enzymes known as glycoside hydrolase enzymes have been identified in the infant gut, which supports this hypothesis. The resulting degraded HMOs can also be used as growth substrates for other infant gut bacteria present in a microbe-microbe interaction known as ‘cross-feeding’. This review describes the current knowledge on HMO metabolism by particular infant gut-associated bacteria, many of which are currently used as commercial probiotics, including the distinct strategies employed by individual species for HMO utilisation.

## Teaser

This review describes the current knowledge on HMO metabolism by a range of infant gut-associated bacteria, many of which are currently used as commercial probiotics, including the distinct strategies employed by individual species for HMO utilisation.

## Introduction

Human breast milk contains a diverse array of essential macro and micronutrients critical for infant health, growth and development. In addition to these nutrients, a number of bioactive compounds and immune factors such as immunoglobulins, growth factors, lysozyme, microRNAs, antibacterial peptides, lactoferrin, and human milk oligosaccharides (HMOs) are present. These components engage in a wide range of biological activities including (i) maturation of the gastrointestinal (GI) tract, (ii) immune modulation, (iii) energy homeostasis, (iv) protection against bacterial and viral pathogens (Lemas et al. 2016), and (v) gut microbiome establishment (Kavanaugh et al. 2013, Morrin et al. 2019). Human breast milk is also a source of beneficial bacteria (e.g. bifidobacteria and lactobacilli) some of which are recognised for their health-promoting benefits. The infant gut microbiome is constantly evolving with its human host from birth onwards, with significant changes occurring during the first three years of life (Zhang et al. 2022). The initial formation and development of the infant microbiome is influenced by a variety of factors such as delivery mode, maternal health status, antibiotic usage and genetic factors (Linehan et al. 2022). Another important factor is the manner of infant feeding, this being accomplished either by breast milk, which provides mother-specific HMOs and reportedly seeds the infant gut with HMO-utilising bacteria (Liu et al. 2022, Zhang et al. 2022) and/or formula milk where breast feeding is not possible.

**Figure 1.**
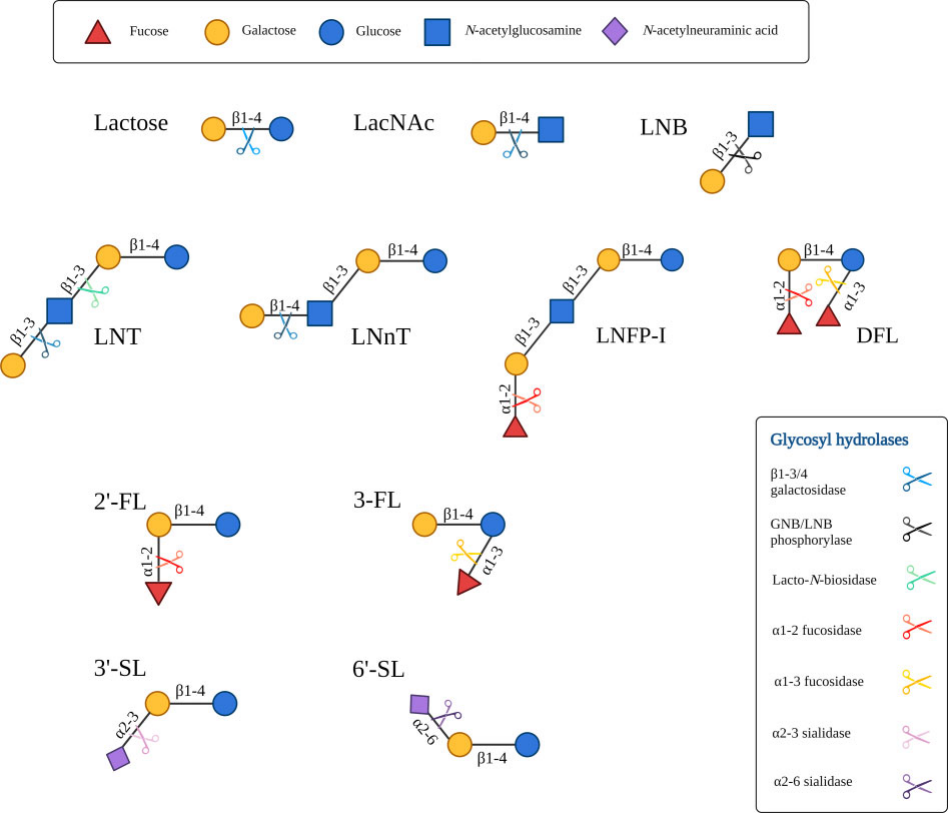
**HMO structures found in human milk**. Structures of the core human milk oligosaccharides (HMOs) found in human milk including linkages of HMO building blocks and the glycoyl hydrolase enzymes which act to degrade HMOs. Legend for the monosaccharide constituents of HMOs on the top and glycosyl hydrolases on the bottom left. HMO and HMO components shown include Lac: lactose; LacNAc: *N*-acetyllactosamine; LNB: lacto-*N*-biose; LNT: lacto-*N*-tetraose; LNnT: lacto-*N*-neotetraose; LNFPI: lacto-*N*-fucopentaose-I; DFL: difucosyllactose; 2’-FL: 2’ fucosyllactose; 3-FL: 3-fucosyllactose; 3’-SL: 3’sialyllactose; 6’-SL: 6’sialyllactose.

HMOs are the third most abundant constituent in human milk following lactose and lipids, being present at a concentration of ∼17 g/L in colostrum, ∼13 g/L in transitional milk, and decreasing to ∼11 g/L in mature milk (Soyyılmaz et al. 2021). The oligosaccharide content of human milk is more diverse and more concentrated than the milk of other mammals, with over 200 distinct HMO structures identified to date (Petschacher and Nidetzky 2016). HMOs are typically composed of five monomers, namely glucose (Glc), galactose (Gal), *N*-acetylglucosamine (GlcNAc), fucose (Fuc) and *N*-acetylneuraminic acid (NeuAc). Fig. 1 outlines the structures of select HMOs and HMO components. HMOs can be linear or branched, with all structures containing a lactose (Lac) core at the reducing end that can be extended by lacto-*N*-biose (LNB) or *N*-acetyllactosamine (LacNAc), which further classifies HMOs as type I or type II, respectively, with type I predominating in human milk (Ruhaak and Lebrilla 2012). Moreover, HMOs can be classified as acidic, neutral or neutral fucosylated, with acidic HMOs containing sialic acid, while fucosylated HMOs contain Fuc residues (Urashima et al. 2012). Fucosylated HMOs can account for up to 70% of all HMOs in human milk and are determined by the mother's secretor and Lewis blood group status (Bode and Jantscher-Krenn 2012). Sialylated HMOs account for just 10%–15% of total oligosaccharide content in human milk, (Lis-Kuberka and Orczyk-Pawiłowicz 2019), unlike bovine milk of which 70% of the oligosaccharides present are sialylated (Martin-Sosa et al. 2003). HMOs are largely resistant to enzymatic digestion in the human digestive tract, allowing them to reach the distal GI tract relatively intact where they can be metabolised by resident bacteria or excreted in faeces (Kunz et al. 2000). Some bacteria which have been shown to utilise HMOs to varying degrees for growth include *Bifidobacterium* and *Lactobacillus* as well as *Akkermansia muciniphilia, Bacteroides* and certain S*taphylococcus* species.

**Figure 2.**
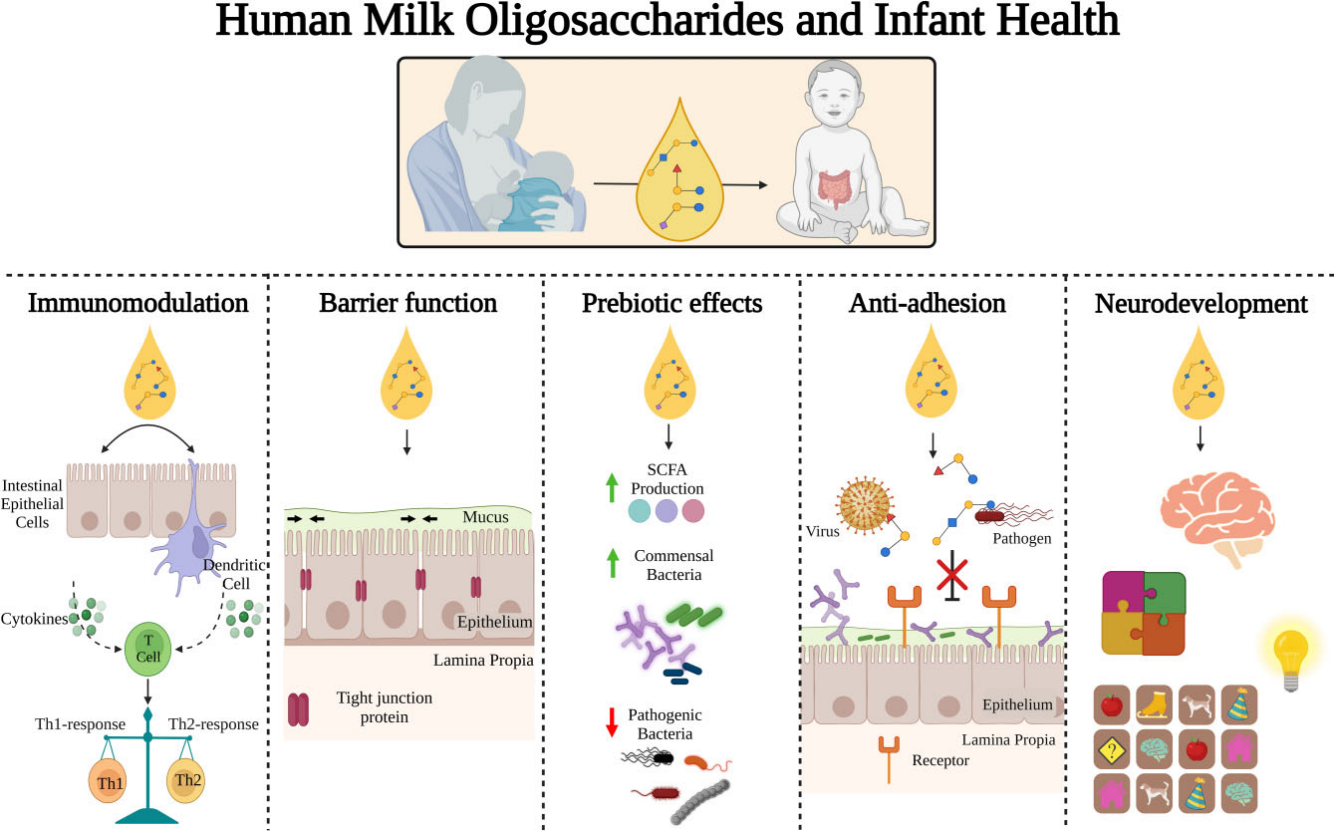
**Functions of HMOs in infant health**. The role of HMOs in the infant gut. HMOs interact with immune cells and can function in immunomodulation by affecting the expression of pro/anti-inflammatory cytokines. HMOs improve intestinal barrier functioning via increasing the expression of tight junction proteins in order to decrease intestinal barrier permeability. HMOs exert prebiotic effects stimulating the growth and colonization of bifidobacteria and other commmensal bacteria in the gut over pathogenic bacteria leading to the production of favourable metabolites such as short chain fatty acids (SCFAs). HMOs also act as anti-virals and anti-microbials by directly binding to viruses and pathogenic bacteria preventing them from adhering to intestinal epithelial cells blocking their infective capacity. Finally, HMOs also have a role in infant neurodevelopment and have been linked with improved learning and memory.

Despite presenting no direct nutritional value, HMOs have been shown to impart numerous benefits to the neonate which are summarised in Fig. 2. HMOs confer prebiotic effects, meaning that they encourage growth and colonization of certain beneficial or ‘probiotic’ bacteria in the gut. Probiotics are defined by the FAO/WHO as ‘live micro-organisms which when administered in adequate amounts confer a health benefit on the host’ (Gibson et al. 2017). HMO utilisation may lead to increased production of favourable metabolites such as short chain fatty acids including acetate and butyrate. Benefits often associated with acetate production include intestinal and immune system development with the latter associated with the release of both anti- and pro-inflammatory cytokines by intestinal cells (Underwood et al. 2014). Acetate may also function as a carbon source to support growth and metabolism of butyrate-producing microorganisms. Additional roles associated with HMOs include anti-microbial activity against bacterial and viral pathogens through their action as molecular decoys thereby preventing attachment to epithelial cells (Garrido et al. 2012, Morrin et al. 2021). Decoy activity is based on the structural resemblance between HMOs and the glycan receptors targeted by pathogens to attach to epithelial cells (Laucirica et al. 2017). Furthermore, HMOs can improve barrier function by promoting increased expression of tight junction proteins to reduce permeability of the intestinal epithelial cell layer, thereby preventing gut dysfunction (Chleilat et al. 2020). HMOs also modulate epithelial cell responses and stimulate immune system development by promoting maturation of dendritic cells, increasing anti-inflammatory cytokines and decreasing the presence of pro-inflammatory cytokines, thus playing a role in maintaining immune system homeostasis (Singh et al. 2022). HMOs can also help quell an overactive T helper cell 2 (Th2) response, resulting in a more balanced Th1/Th2 profile and thereby alleviating a sustained overreactive immune response that may otherwise result in the emergence of an allergic state (Rousseaux et al. 2021) Certain HMOs have also been shown to play a role in neurodevelopment and cognition, and have been associated with improved learning and memory (Willemsen et al. 2023).

Over the past 50 years, substantial efforts have been made by various research groups around the world to unravel the unique composition of human milk. Advances in breast milk research has led to advances in infant milk formulation and applications, resulting in the use of bioactives such as biosynthetically produced HMOs and probiotics, predominantly 2’-fucosylactose and *Bifidobacterium* and/or *Lactobacillus* spp., respectively. Industrial scale production of HMOs is possible primarily due to the use of genetically modified micro-organisms which can overexpress recombinant enzymes for the production of specific HMO structures. Thus, many infant formulas (IF) are now enriched with 2’-fucosyllactose (2’-FL) and lacto-*N*-neotetraose (LNnT), two of the most abundant HMOs found in breast milk, following EU approval for novel food status (Commission Implemented Regulation (EU) 2017/2470) (Vandenplas et al. 2018). In addition, lacto-*N*-tetraose (LNT), 3-fucosyllactose (3-FL) and 3’-sialyllactose (3’-SL) have been granted EU authorisation to be placed on the market for safe use in infant formula as of 2023 (Commission Implementing Regulation (EU) 2023/7, 2023/52 and 2023/113 respectively (Amending Commission Implemented Regulation 2017/2470).

Many excellent scientific reviews focus on the structure and biological functions as well as the concentrations of HMO in human milk throughout lactation and the endogenous synthesis of HMOs (Singh et al. 2022, Hu et al. 2023, Zhu et al. 2023). In the current review, we will focus on HMO metabolism and describe the current knowledge on HMO-metabolizing abilities of certain infant gut-associated bacteria such as *Bifidobacterium* and *Lactobacillus* as well as *Akkermansia muciniphilia* and *Bacteroides* species. The strategies employed by individual species which utilise HMOs as substrates will be discussed which in turn should shed light on the reasons behind the prevalence of certain species over others in the infant gut microbiome.

## HMO enzymatic degradation by glycoside hydrolase enzymes

The structure-specific degradation of HMOs requires a complex repertoire of enzymes known as glycoside hydrolases (GHs), which function to cleave glycosidic bonds between individual HMO structures (Fig. 1) in order to release constituent monosaccharides or disaccharides as a prelude to further metabolism (Garrido et al. 2013). Certain bacteria, particularly bifidobacterial species such as *Bifidobacterium longum* subsp. *infantis* and *Bifidobacterium bifidum*, possess an abundance of genes that encode enzymes involved in the metabolism of host glycans such as HMOs (Milani et al. 2015). It should be noted that the presence alone of genes associated with HMO degradation does not however guarantee the production of active enzymes which function to degrade HMO structures. Functional analysis of breast-fed infant microbiomes has shown an enrichment of these enzymes and expression of genes related to HMO degradation (Bäckhed et al. 2015, Milani et al. 2015). GH enzymes which are directly involved in HMO metabolism include fucosidases, sialidases, β-galactosidases, β-*N*-acetylhexosaminidases and lacto-*N*-biosidases, all of which function to degrade a diverse array of HMO structures including sialylated, neutral and neutral fucosylated HMOs. These gut microbiome-associated enzymes are essential for HMO utilisation by infant-associated gut bacteria and the location of these enzymes can be either intracellular or extracellular depending on the particular species. An understanding of the mechanism of action of these enzymes aids in revealing which and how HMO structures are degraded (Zhang et al. 2022). Table 1 summarises the various enzyme families required to metabolise HMOs as compiled from a number of well characterised infant associated strains.

**Table 1.** Glycosyl hydrolase enzyme families required to metabolise HMOs and their distribution amongst well characterised species. Strains described are a combination of type strains where available or strains which are included in the Carbohydrate active enzymes (CAZy) database.

			GH2	GH20	GH29	GH33	GH42	GH95	GH112	GH136
			β-galactosidase	β-hexosaminidase	Fucosidase	Sialidase	β-galactosidase	Fucosidase	GNB/LNB Phosphorylase	Lacto*-N*-biosidase
Phylum	Species	Strain	Number of enzymes present in genome							
**Actinobacteria**	*Bifidobacterium longum* subsp. *infantis*	ATCC 15 697	3	3	3	2	3	1	1	0
	*Bifidobacterium longum* subsp. *longum*	DJO10A	2	1	0	0	3	0	1	0
	*Bifidobacterium bifidum*	PRL 2010	3	4	1	2	2	1	2	1
	*Bifidobacterium breve*	UCC 2003	5	1	0	1	2	1	1	0
	*Bifidobacterium catenulatum* subsp. *kashiwanohense*	APCJK1	4	1	2	0	3	1	0	0
**Firmicutes**	*Lacticaseibacillus casei*	BL23	0	1	3	0	0	0	0	1
	*Lacticaseibacillus rhamnosus*	GG	3	0	3	0	0	0	0	0
	*Lactiplantibacillus plantarum*	WCFS1	1	1	0	0	1	0	0	0
	*Lacticaseibacillus paracasei*	JCM 8130	0	1	1	0	0	0	0	0
	*Lactobacillus helveticus*	LH99	1	0	0	0	1	0	0	0
	*Lactobacillus acidophilus*	LA-14	1	0	0	0	2	0	0	0
**Bacteroidetes**	*Bacteroides thetaiotaomicron*	DSM 2079	31	14	9	2	1	5	0	0
	*Bacteroides fragilis*	NCTC 9343	15	12	9	3	0	3	0	0
**Verucomicrobia**	*Akkermansia muciniphilia*	Amuc	6	11	4	2	0	2	0	0

Fucosylated or sialylated HMO structures require removal of the sialic acid and fucose substitutions prior to the metabolism of the core structure (Ashida et al. 2009), which generally results in the release of monosaccharides and disaccharides (Kitaoka 2012, Zhang et al. 2022). Enzymes which release Fuc from fucosylated HMOs are termed fucosidases and belong to the GH29 (EC 3.2.1.51, EC 3.2.1.111 and EC 3.2.1.63) (Carbohydrate Active Enzymes Database http://www.cazy.org/) and GH95 families (EC 3.2.1.51 and EC 3.2.1.63) (Drula et al. 2022). These include 1,2-α-fucosidase enzymes from the GH95 (EC 3.2.1.63) family, which act on α-1,2 linkages, while 1,3/4-α-fucosidase enzymes (EC 3.2.1.111) act on fucosylated HMOs with α-1,3 or α-1,4 linkages. The specificities of these enzymes also differ, with the 1,2-α-fucosidase acting mainly on 2’-FL and lacto-*N*-fucopentaose I (LNFP I) (Katayama et al. 2004), while the 1,3/4-α-fucosidase has a preference for structures such as 3-FL and lacto-*N*-fucopentaose II/lacto-*N*-fucopentaose III (LNFP II/III) (Ashida et al. 2009). With regards to sialylated oligosaccharides, sialidase enzymes from the GH33 (EC 3.2.1.18) family act on α-2,3 and α-2,6 linkages, such as those found in 3’-SL and 6’-sialylactose (6’-SL) respectively, and are responsible for the liberation of sialic acid (Neu5Ac) from the core structure (Masi and Stewart 2022).

Following the removal of the Fuc or sialic acid, the core HMO structure is now accessible for further degradation (Masi and Stewart 2022). This core HMO structure can either be type I or type II as mentioned above with type I HMOs containing a Lac core, which can be elongated by lacto-*N*-biose (Galβ1–3GlcNAc; LNB) resulting in LNT, while type II HMOs contain a Lac core coupled to an *N*-acetyllactosamine (LacNAc; Galβ1–4GlcNAc) unit which together can form lacto-*N*-neotetraose (LNnT) (Urashima et al. 2012). Hydrolysis of the β1-3 linkage of LNB in type I HMOs and the β1-4 connection of LacNAc in type II HMOs, is catalysed by β1-3 galactosidase from the GH42 (EC 3.2.1.23) family and a β1-4 galactosidase from the GH2 (EC 3.2.1.23) family respectively, releasing Gal and lacto-*N*-triose II (LNTri II). The released LNTri II can then be degraded by β-hexosaminidases/β-1,6- *N*-acetylglucosaminidases from the GH20 (EC 3.2.1.-) family (Ioannou et al. 2021). Lacto-*N*-biosidase from the GH136 family can also act on β1-3 linkages in LNT to generate LNB and Lac which can be further metabolised (Sakurama et al. 2013, Masi and Stewart 2022). Any remaining Lac present either from the metabolism of HMOs or free Lac from milk can then be utilised by β-galactosidases from the GH2 (EC 3.2.1.23) and GH42 (EC 3.2.1.23) families which hydrolyse β1-4 linkages such as those found in Lac (Ambrogi et al. 2021, Ioannou et al. 2021).

**Figure 3.**
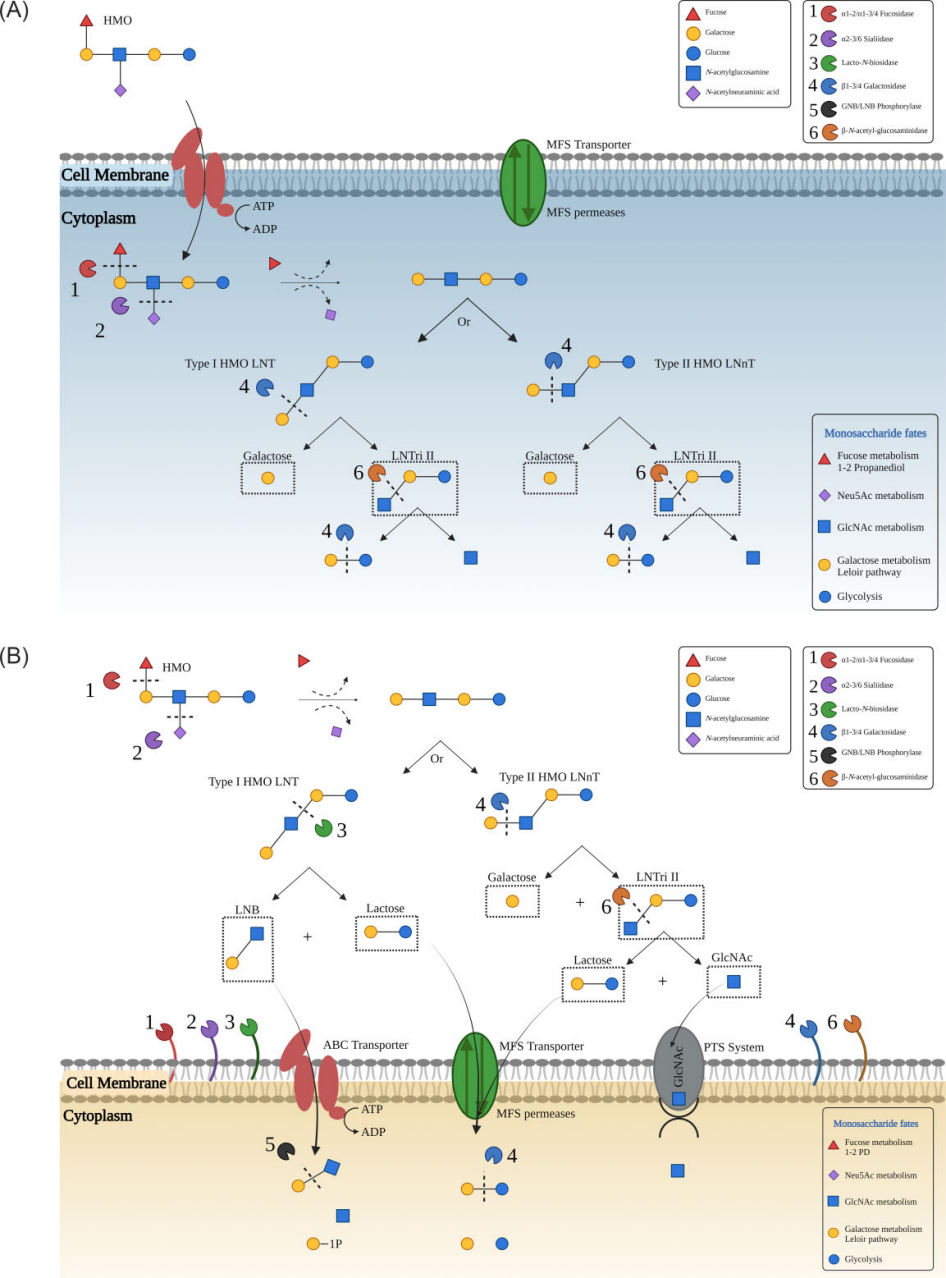
**(A and B) HMO degradation strategies used by infant asociated bacteria**. Possible strategies infant associated bacteria utilise for the consumption of HMOs. Intracellular HMO degradation (3a) involves the transportation of intact HMOs directly into the cells via the action of transporters where inside the cells HMOs are hydrolysed into monosaccharide components by glycosyl hydrolases enzymes present in the cytoplasm. Extracellular HMO degradation (3b) in contrast employs cell-wall anchored glycosyl hydrolase enzymes in order to degrade HMOs extracellularly resulting in the formation and release of mono and disaccharide sugars, which can then be transported intracellularly to be metabolised inside the cell. The fates of the subsequent monosaccharides is also highlighted in the images.

HMO metabolism by GHs can occur either intracellularly (Fig. 3a) or alternatively their degradation can be extracellular (Fig. 3b). Intracellular HMO utilisation is common in various bifidobacterial species, in particular *Bifidobacterium longum* subsp. *infantis*, and has also been suggested for certain lactobacilli. Intracellular HMO degradation typically involves transportation of HMOs directly into a cell by ATP binding cassette (ABC) transporters where they are hydrolysed into their constituent monosaccharides by GHs in the cytoplasm (Kitaoka 2012, Sakanaka et al. 2019b). The second method which is employed in order to utilise HMO includes an extracellular degradation strategy whereby cell-wall anchored, glycoside hydrolases act to hydrolyse HMOs extracellularly resulting in the formation and subsequent release of mono and disaccharide sugars which can function to promote the growth of other bacteria or be transported internally to be metabolised inside the cell (Kitaoka 2012, Zhang et al. 2022). The method of HMO degradation which occurs is completely dependent on the individual species as will be discussed below.

## HMO metabolism at species level

### HMO Metabolism by bifidobacterial species

Bifidobacteria are the predominant breast-fed infant-associated bacteria capable of metabolising human milk-associated prebiotic components, in particular HMOs. Certain *Bifidobacterium* species are well adapted for this function, a point which is illustrated by the presence of a number of bifidobacterial species at both a high prevalence and abundance in the stools of healthy breast-fed infants (Milani et al. 2017, James et al. 2019). The ability to metabolise specific HMOs is most often associated with a select number of *Bifidobacterium* species, including *Bifidobacterium longum* subsp. *infantis* (*B. infantis*), *Bifidobacterium bifidum* (*B. bifidum*)*, Bifidobacterium breve* (*B. breve*)*, Bifidobacterium longum* (*B. longum*), *B. catenulatum* subsp. *kashiwanohense (B. kashiwanohense)* and *B. catenulatum*. Carbohydrate metabolic capabilities differ significantly between *Bifidobacterium* species with *B. infantis* and *B. bifidum* often described as avid HMO consumers, based on their genetically encoded ability to produce glycoside hydrolase enzymes required to catabolise an array of HMO linkages. *B. breve* and *B. longum* on the other hand are often more limited and restricted to certain and less complex HMOs such as LNT or LNnT, or HMO-specific components such as sialic acid and Fuc through cross-feeding (Gotoh et al. 2019, Ioannou et al. 2021). Cross-feeding is an important microbe-microbe interaction whereby carbohydrate components which are released as a result of the extracellular activities of bacteria present in the intestine are made available as substrates for other members of the gut microbiome. This activity has numerous benefits including increased interactions between members of the gut microbiome as well as an increased production of favourable metabolites (Turroni et al. 2018, Smith et al. 2019, Zhu et al. 2023).

Bifidobacteria dedicate approximately 13.7% of their genome content to carbohydrate metabolism (Milani et al. 2014). As previously mentioned, there are two main strategies which are employed by bifidobacteria in order to degrade HMO structures, intracellular and extracellular degradation (Fig. 3). HMO degradation is a species specific ability with *B. infantis* and *B. breve* strains commonly associated with intracellular degradation whereby oligosaccharide transporters are employed in order to import intact HMOs into the cell which are metabolised into their monosaccharide components. This substantially differs from the extracellular degradation strategy which is most commonly observed for *B. bifidum* strains (Turroni et al. 2010). The strategy of HMO degradation used by *B. longum* strains is dependent on the presence or absence of extracellular fucosidase and sialidase enzymes as well as the gene (*lnbX*) encoding lacto-*N*-biosidase enzyme (LnbX) with strains harbouring *lnbX* gene utilising extracellular glycosidases while those which are negative for *lnbX* instead require oligosaccharide transporters to internalise HMOs (Gotoh et al. 2018). The following sections will discuss HMO metabolism by individual *Bifidobacterium* species.

#### *Bifidobacterium longum* subsp. *infantis*

*Bifidobacterium infantis* is often considered to be the predominant HMO utilising species in the infant gut (Sela and Mills 2014). This property is conserved across the entire subspecies, with numerous studies demonstrating the ability of *B. infantis* strains to grow significantly on a wide range of distinct HMOs including 2’-FL, 3-FL, LNT, LNnT, 3’-SL, 6’SL, LNFP-I, and LDFT (lactodifucotetraose) as the sole carbon source, exceeding the HMO catabolic capacity of other bifidobacterial species, including *B. longum* subsp. *longum, B. breve* and *B. adolescentis* isolates (Sela et al. 2008, Locascio et al. 2009, Cheng et al. 2020b, Ojima et al. 2022). While HMO metabolism is a conserved phenomenon observed in *B. infantis*, HMO metabolic capabilities can vary significantly between strains in terms of preferential utilisation of specific HMO structures as shown through variability in growth kinetics amongst *B. infantis* strains in many studies (Duar et al. 2020, Lawson et al. 2020, Walsh et al. 2022). *B. infantis* strains possess a 43-kb gene cluster specific for HMO degradation (Sela et al. 2008). This gene cluster encodes all GH enzymes required to efficiently cleave HMOs including 1,2-α-fucosidases belonging to the GH95 family, 1,3/4-α-fucosidases from GH29, 2,3/6 sialidases belonging to the GH33 family, β-*N*-acetylhexosaminidase enzymes from the GH20 family, β-galactosidases from the GH2 family and LNT β-galactosidases from the GH42 family (Sela et al. 2008, Garrido et al. 2012, Kitaoka 2012). Interestingly, these enzymes appear to be intracellular due to the absence of an N-terminal signal sequence in their primary structure (Sela et al. 2008). This gene cluster also includes several genes which encode sugar transporters involved in importing HMOs intact such as ATP-binding cassette (ABC) transporters, GNB/LNB pathway transporters as well as solute binding proteins (SBPs) (Garrido et al. 2011). SBPs have a high affinity for specific HMOs or HMO components and have been shown to be exclusively induced during exponential growth on HMOs, with some of these proteins shown to bind epithelial surfaces *in vitro*. This phenomenon is possibly due to structural similarities between HMOs and epithelial glyco-conjugates and the interaction of SBPs with epithelial cell surfaces has been shown to enhance the production of anti-inflammatory cytokines and tight junction proteins (Chichlowski et al. 2012). As mentioned, HMO utilisation by *B. infantis* appears to be intracellular. *B. infantis* strains internalize a range of HMOs such as LNT, LNnT and LNB, fucosylated HMOs including 2’-FL and 3-FL and sialylated HMOs such as 3’-SL and 6’-SL. These structures are then sequentially degraded for subsequent metabolism of the generated monosaccharides by exo-glycosidases in the cytoplasm (Kitaoka 2012).

Whole genome sequencing of *B. infantis* ATCC 15697 confirmed the presence of a number of genes encoding α-L-fucosidases which are involved in hydrolysing an array of glycosidic linkages found in fucosylated HMOs. These genes are located on HMO cluster I and three of the corresponding fucosidases belong to the GH29 family (Blon_0248, Blon_0426 and Blon_2336) with the remaining fucosidases being members of the GH95 (Blon_2335) and GH151 families (Blon_0346) (Sela et al. 2008, Sela et al. 2012). Blon_2335 has been shown to be a particularly efficient α-1,2 fucosidase which is also considerably active on α-1,3 and α-1,4 fucosyl linkages. Blon_2336 is more selective and is specific for α1,3/4 linkages including those present on 3-FL and LNFP III (Sela et al. 2012). *B. infantis* strains also readily consume sialylated HMO structures such as 3’-SL and 6’-SL with *B. infantis* ATCC 15697 expressing two sialidases belonging to the GH33 family which are capable of degrading sialyllactose denoted as NanH1and NanH2 (Kiyohara et al. 2011). NanH2 is an intracellular α-sialidase enzyme which removes sialic acid from α-2,3 and α-2,6 sialyl linkages found in selected HMOs such as disialyllacto-*N*-tetraose (DSLNT) (Sela et al. 2011).

There are two β-galactosidase enzymes which work intracellularly to metabolise short HMOs in *B. infantis* strains. The first β-galactosidase in this gene cluster (Bga2A) encoded by Blon_2334, belongs to the GH2 family and has a preference for Lac, selectively hydrolysing type II but not type I sugars acting on LNnT preferentially (Yoshida et al. 2012). Studies have shown that *B. infantis* strains possess an additional β-galactosidase (Bga42A) encoded by blon_2016 belonging to the GH42 family. This enzyme is thought to exclusively utilise short type I HMOs due to its high specificity for LNT, hydrolysing LNT up to 40 times more efficiently than LNB, Lac and type II HMOs (Asakuma et al. 2011, Yoshida et al. 2012). Free Lac can also be hydrolysed by β-galactosidase enzymes (Garrido et al. 2012) into Glc and Gal which then can enter the Leloir and fructose-6-phosphate (F6P) phosphoketolase (bifid shunt) pathways. These are the major metabolic pathways for D-galactose catabolism into glucose-1-phosphate and for the phosphorolysis of fructose-6-phsphate using the fructose-6-phosphoketolase enzyme (EC 4.1.2.2), respectively (Caputto, Leloir and et al. 1949, de Vries and Stouthamer 1967, Pokusaeva et al. 2011).

The metabolism of HMOs by *B. infantis* leads to the production of host utilisable short chain fatty acids (SCFAs), including acetate and butyrate as well as the organic acid, lactic acid. These may be converted into butyrate by the presence of other gut commensals. Therefore, HMOs simulate bifidobacterial growth and by cross-feeding, increase the production of butyrate, an important energy source for colonocytes (Koh et al. 2016). A pathway for the anaerobic catabolism of L-fucose via non-phosphorylated intermediates has been described for *B. infantis* ATCC 15697, which results in the formation of the propionate precursor 1,2-propanediol (1,2-PD) during growth on fucosyllactose, which can be utilised by cross-feeding to produce propionic acid. This mechanism has also since been demonstrated in *B. breve, B. kashiwanohense* and *B. longum* subsp. *suis* suggesting a common pathway for the metabolism of L-fucose present amongst these species (Bunesova et al. 2016, Schwab et al. 2017, James et al. 2019).

#### Bifidobacterium bifidum

*Bifidobacterium bifidum* contains all of the glycoside hydrolases required to catabolise an array of HMO linkages, allowing this species to serve as a primary degrader of these complex glycans (Garrido et al. 2013). *B. bifidum* strains are known to use a number of extracellular, cell wall anchored glycoside hydrolases in order to metabolise HMOs into their respective monosaccharides and disaccharides (Kitaoka 2012). Studies examining the liberation of LNB from HMOs in *B. bifidum* JCM1254 have shown utilisation of an extracellular enzymatic system, with seven key enzymes playing a role in metabolism of HMOs (Kitaoka 2012). First, two α-fucosidase enzymes are required to cleave HMOs, with these acting on fucosylated HMOs such as 2’-FL, 3-FL, LNFP I/II/III and LNDFH I/II in order to reveal the core structures of the HMOs (Asakuma et al. 2011). The first of these α-fucosidase enzymes, 1,2-α-fucosidase (AfcA) which belongs to the GH95 family, hydrolyses the fucosyl unit bound to the second position of the galactosyl residue found at the non-reducing end of HMOs and is involved in the utilisation of 2’-FL (Katayama et al. 2004). The second α-fucosidase, 1,3/4-α-fucosidase (AfcB), belongs to the GH29 family and is required for hydrolysis of the fucosyl unit neighbouring the β-linked galactose and releases α-1,3/4 fucosyl residues from substrates such as 3-FL and LNFP II/III into the growth medium, where Fuc can be utilised by other gut microbes present (Ashida et al. 2009).

There are two 2,3/6-α-sialidases belonging to GH33 which are involved in HMO metabolism in *B. bifidum*, SiaBBI and SiaBBII (Kitaoka 2012, Nishiyama et al. 2017). SiaBBI and SiaBBII enzymes remove sialic acid residues from HMOs, promoting degradation of sialylated oligosaccharides (Nishiyama et al. 2017). These sialidases contain an N-terminal signal sequence, a sialidase catalytic domain and a C-terminal transmembrane region indicating that these are extracellular, membrane anchored enzymes (Kiyohara et al. 2011). Another extracellular enzyme which is crucial in the metabolism of HMOs in *B. bifidum* is lacto-*N*-biosidase (LnbB) which belongs to the GH20 family. The *lnbB* gene encodes a protein which contains a signal peptide at the N-terminus and a membrane anchor at the C-terminus (Wada et al. 2008). There is also a β-galactosidase belonging to the GH2 family known as BbgIII as well as a β-*N*-acetylglucosaminidase, BbhI which belongs to the GH20 family which allow *B. bifidum* to digest type II HMOs (Miwa et al. 2010).

#### *Bifidobacterium longum* subsp. *longum*

*Bifidobacterium longum* has been repeatedly shown to predominate the breast-fed infant gut microbiome (Laursen et al. 2021). Subspecies of *B. longum* include *B. longum* subsp. *infantis, B. longum* subsp. *longum* and *B. longum* subsp. *suis* (Sakata et al. 2002, Mattarelli et al. 2008). Despite originating from the same species, these subspecies differ greatly in their HMO utilisation abilities. For instance, *B. longum* subsp. *infantis* is a particularly avid consumer of HMOs (see above), while HMO utilisation by *B. longum* subsp. *longum* strains is typically limited to LNT and LNB (Ward et al. 2007, Asakuma et al. 2011) and the ability to utilise any other HMO such as 2’-FL, 3-FL, LNnT, and LNFP I/II/III has only been reported for a select number of strains (Gotoh et al. 2018). Comparative genomic hybridisation (CGH) analysis has determined the HMO utilisation gene regions such as those which contain fucosidases, sialidases, ABC transporters and SBPs, all of which are needed for efficient metabolism of HMOs, are conserved across *B. longum* subsp. *infantis* and *B. bifidum* strains capable of HMO utilisation and have diverged and are absent in *B. longum* subsp. *longum* strains which often do not display significant growth on HMOs (LoCascio et al. 2010). This points to a specialisation of specific *Bifidobacterium* species for the utilisation of milk oligosaccharides.

The inability of many *B. longum* strains to readily consume specific HMOs is due to its lack of specific GH enzymes required to facilitate this metabolism. For example, studies examining HMO metabolism by *B. longum* DJO10A found that it lacks fucosidases belonging to the GH29 and GH95 families, or sialidase members of the GH33 family which are required for the consumption of specific types of fucosylated and sialylated HMOs (Marcobal et al. 2010). This strain does however possesses genes encoding β-galactosidase enzymes from the GH2 and GH42 families and is therefore capable of metabolising some low molecular weight HMOs such as LNT and LNB, consuming LNT from pooled HMOs (Marcobal et al. 2010). Garrido *et al*. examined the growth ability of 17 *B. longum* strains on pooled HMOs and on individual structures such as LNT, LNnT, 2’-FL, 3-FL, 3’-SL and 6’-SL. These HMOs were used as the sole carbohydrate source and were present at a concentration of 2%. Growth was evaluated by measuring optical density at a wavelength of 600 nm which is commonly used to determine cell density in liquid cultures, with 16 of the 17 strains examined reaching an OD600 of 0.2–0.5 following 24 hours growth on HMO. In addition, 15 of the 17 strains displayed significant growth on 2% LNT as the sole carbohydrate source present (Garrido et al. 2016). *B. longum* SC596 was shown to possess the capacity to utilise fucosylated HMOs such as fucosyllactose in addition to LNT and LNnT. The ability to utilise the fucosylated HMOs, 2’-FL and 3-FL was also reported for *B. longum* SC568 with a total of 30% of fucosylated HMOs consumed (Garrido et al. 2016). This ability is most likely due to the presence of a gene cluster region dedicated to the utilisation of fucosylated HMOs termed the Fucosylated Human Milk Oligosaccharides utilisation (FHMO) cluster which encodes the components necessary for the import of fucosylated structures as well as genes encoding two α-fucosidases. None of the strains tested in this study had the ability to grow on 2% 3’-SL or 6’-SL as the sole carbon source, indicating that *B. longum* strains lack sialidases necessary for metabolism of sialylated HMOs (Garrido et al. 2016).

As mentioned above, a novel gene cluster present in *B. longum* strains, dedicated to the metabolism of fucosylated HMOs termed the FHMO cluster has previously been identified to be present in a small number of *B. longum* strains (Garrido et al. 2016). This FHMO cluster shares significant homology with two ABC transporters present in *B. infantis* ATCC15697 and part of the HMO cluster I which can be found in *B. infantis* (Sela et al. 2008). Fucose metabolism genes and both α-fucosidases from GH29 and GH95 (BLNG_01263 and BLNG_01264) which are necessary for the utilisation of fucosylated HMOs are found on this cluster. BLNG_01263 has been shown to preferentially digest α-1–3/4 fucosyl linkages while BLNG_01 264 was shown to digest α-1–2 fucosyl linkages and certain α-1–3 linkages such as those in 2’-FL, LDFT and LNFP (Garrido et al. 2016). These fucosidases appear to act synergistically, with complementary specificities in order to consume various fucosylated HMOs. It is unsurprising that *B. longum* SC596, one of the few *B. longum* strains which has been shown to utilise fucosylated HMOs, is known to contain this gene cluster on its genome (Garrido et al. 2016). This FHMO cluster appears to be absent in *B. longum* DJO10A as well as *B. longum* LH12, two strains which while capable of supporting some growth on HMOs are unable to utilise fucosylated HMOs highlighting the role of this FHMO cluster in fucosylated HMOs metabolism in certain *B. longum* strains (Lawson et al. 2020).

In *B. longum* SC596, two genes encoding intracellular β-galactosidases have been shown to be expressed, BLNG_00015, belonging to the GH2 family and BLNG_01753, belonging to the GH42 family. BLNG_00015 has a preference for type II HMO linkages specifically β-1–4 linkages releasing Gal from Lac and LNnT while BLNG_01753 is specific for type I HMOs such as LNT and lacto-*N*-hexaose (LNH). These neutral HMOs are all consumed during the late exponential phase, after the preferential consumption of fucosylated HMOs initially by *B. longum* SC596. These enzymes are presumed to be intracellular due to the apparent lack of a transmembrane domain or signal peptide sequence (Garrido et al. 2016). *Bifidobacterium longum* SC596 is therefore capable of utilising neutral HMOs, in a similar manner to *B. infantis* strains, whereby intact HMOs are imported into cells first, followed by intracellular degradation via the activity of various intracellular GHs.

Studies have shown that a gene (*lnbX*), which encodes lacto-*N*-biosidase (LnbX), an important extracellular HMO-degrading enzyme found in *B. longum*, is often enriched in the stool of exclusively breast-fed infants compared to exclusively formula-fed or mixed-fed infants (Kitaoka et al. 2005, Yamada et al. 2017). This *lnbX* gene, is similar to the *B. bifidum lnbB* gene, which encodes the GH20 family enzyme required for the metabolism of LNT, with both LnbX and LnbB enzymes highly specific for LNT hydrolysis. The product of the *lnbX* gene has been shown to be responsible for the hydrolysis of LNT into LNB and Lac (Sakurama et al. 2013).

The absence of *lnbB* and *lnbX* homologs in other *Bifidobacterium* species highlights their unique function in LNT metabolism in *B. longum* strains when compared to *B. breve* and *B. infantis. lnbX* is not ubiquitously found in all *B. longum* strains but is instead strain-dependent with less than half of all *B. longum* genomes sequenced to date containing this gene (Gotoh et al. 2018). Strains which contain this *lnbX* are termed *lnbX* positive and utilise GNB/LNB-BP for uptake of LNB and GNB/LNB phosphorylase for intracellular phosphorolysis while *lnbX* negative strains utilise LNT β-1,3-galactosidase for the intracellular hydrolysis of LNT (Sakanaka et al. 2019a). *B. longum* strains which are *lnbX* deficient have limited ability to assimilate LNT, suggesting *lnbX* is required for the metabolism of LNT in *B. longum* (Sakurama et al. 2013, Yamada et al. 2017).

It is clear that HMO utilisation strategies employed by *B. longum* are variable and strain specific. This phenomenon is similar to what is observed in other *Bifidobacterium* species such as *B. bifidum* and *B. breve*. The preferential utilisation of certain HMOs has been well described and is noted to differ considerably between *Bifidobacterium* species and also between strains. This is evident for *B. longum* as certain strains only utilise LNT and LNB, while selected strains have developed sophisticated strategies in order to metabolise a range of complex structures such as 2’-FL, 3-FL, LNnT, LDFT, and LNFP I/II/III. While *B. longum* strains do not generally exhibit a preference for fucosylated HMOs over neutral HMOs, mass spectrometry based glycoprofiling of HMO consumption using HMOs purified from pooled breast milk has shown that one strain *B. longum* SC596 can have a preference for fucosylated HMOs preferentially utilising these from HMO pools before neutral HMOs are consumed (Garrido et al. 2016).

#### Bifidobacterium breve

*Bifidobacterium breve* is one of the species which is most frequently isolated from the stool of new-borns, despite limited growth on HMOs *in vitro* (Asakuma et al. 2011, Thongaram et al. 2017). It has been demonstrated that *B. breve* functions as a ‘scavenger’ species through cross-feeding on HMO-derived monosaccharides which are released as a result of extracellular hydrolytic activities by other *Bifidobacterium* strains found in the infant gut (Sela and Mills 2010, Egan et al. 2014a, O'Connell Motherway et al. 2018, Cheng et al. 2020a, Walsh et al. 2022). *B. breve* strains have the capacity to utilise certain type I and type II HMOs, however, this ability appears to be strain dependent and subject to variability (Thongaram et al. 2017). At least 55 GH genes have been found on the *B. breve* genomes sequenced to date, with several of these shown to be involved in the metabolism of HMOs (Belkaid and Hand 2014). The HMO consumption mechanisms observed in *B. breve* are broadly similar to those found in *B. longum* subsp. *infantis* whereby oligosaccharides such as LNT, LNnT and LNB are imported first, and then subjected to intracellular degradation and metabolism of their monosaccharide components (James et al. 2016, Sakanaka et al. 2019a). As mentioned, considerable strain-dependent diversity in the ability to grow on HMOs exists among *B. breve* strains with some strains growing vigorously on pooled HMOs, while other strains exhibit limited or poor growth on the same HMO mix (Ruiz-Moyano et al. 2013). The latter cited study examined the ability of six selected *B. breve* strains to consume 22 different oligosaccharides during growth on a HMO pool and demonstrated that HMO utilisation varied among these strains with a range of 23–42% of the HMOs consumed as quantified using nano-HPLC-chip/TOF MS (Ruiz-Moyano et al. 2013).

A well characterised *B. breve* strain, *B. breve* UCC2003 encodes a number of carbohydrate-modifying enzymes enabling this strain to utilise certain HMOs or HMO-derived carbohydrates (James et al. 2016). Genome sequencing of *B. breve* UCC2003 confirmed the presence of an intracellular β-*N*-acetylhexosaminidase from the GH20 family, denoted *nahA*, which can liberate GlcNAc from lacto-*N*-triose II (LNTri II) (James et al. 2016). *Bifidobacterium breve* strains have also been shown to express β-galactosidase enzymes which function to hydrolyse type II HMOs, and can liberate Gal from LNT and LNnT (Asakuma et al. 2011). A β-galactosidase from the GH42 family is also present on the genome of *B. breve* UCC2003 (encoded by Bbr_0529) designated *lntA*, which functions in the metabolism of GNB/LNB (James et al. 2016). A second β-galactosidase from the GH2 family designated *lacZ2* (Bbr_0010) is able to hydrolyse LNnT, releasing Gal and LNTri II. This LNTri II is then hydrolysed by *nahA*, liberating Lac and GlcNAc, with the Lac then further broken down by β-galactosidase enzymes releasing Glc and Gal (O'Connell Motherway et al. 2013, James et al. 2016). Bbr_1587, denoted *lnbP*, encodes a homolog of LNBP from the 1,3- β-galactosyl-*N*-acetylhexosamine phosphorylase family (GH112) (Xiao et al. 2010), which is predicted to function in the cleavage and phosphorylation of LNB as well as its entry into the GNB/LNB pathway (Nishimoto and Kitaoka 2007). This *lnbP* gene is found in the Bbr_1585–1590 cluster along with genes which encode a UDP-glucose-4-epimerase, a phosphotransferase family protein, two permease proteins and a solute-binding protein which are all hypothesised to function in the metabolism of GNB/LNB by UCC2003 (James et al. 2016).

*Bifidobacterium breve* is thought to selectively utilise certain HMOs such as LNT, LNnT and LNB, but assimilation of other HMOs is limited, variable and strain dependent (Asakuma et al. 2011, Sela 2011, Kitaoka 2012, Walsh et al. 2022). While it is not true for the majority, some *B. breve* strains are capable of degrading 2’-FL and 3-FL (Ruiz-Moyano et al. 2013, Bunesova et al. 2016). The majority of *B. breve* strains are known to contain an intracellular 1,2-α-fucosidase from the GH95 family, while only selected strains contain a second GH29 1,4-α-fucosidase enzyme. Growth of *B. breve* on fucosylated HMOs such as 2’-FL and 3-FL is strain-dependent despite the presence of a putative fucosidase-encoding gene. Therefore, it appears that the presence of this second GH29, 1,4-α-fucosidase determines the ability of certain *B. breve* strains to utilise 2’-FL. Studies examining the growth of *B. breve* strains on 2% 2’-FL found that strains which were lacking the gene encoding this second GH29 enzyme, failed to grow on 2’-FL as the sole carbon source (Ruiz-Moyano et al. 2013). Strains of *B. breve* which possess this second α-fucosidase-encoding gene displayed significant growth on 2’-FL as well as some limited growth on 3-FL as the sole carbon sources present (Ruiz-Moyano et al. 2013). It should be noted however that some strains which do possess this additional 1,4-α-fucosidase-encoding gene are unable to grow on 2’-FL. The authors suggest that this may be due to a lack of gene induction or an inability to import the 2’-FL substrate (Ruiz-Moyano et al. 2013).

Growth on both 3’-SL and 6’-SL is uncommon in *B. breve* strains and was only observed in one of the 24 strains examined in a comprehensive study evaluating growth on these HMOs by measuring optical density at 600 _nm_ (Ruiz-Moyano et al. 2013). Limited ability of one particular *B. breve* strain, *B. breve* M-16 V to utilise both 3’-SL and 6’-SL from a pool representing HMOs found in secretor breast milk has recently however been observed (Walsh et al. 2022). The ability of *B. breve* strains to utilise sialic acid however is well documented and can involve the GH33 family exo-sialidase which allows bacteria to utilise host mucins to liberate sialic acids (Egan et al. 2014b, Lawson et al. 2020). Many *B. breve* strains are reported to possess the gene encoding this α-sialidase (Ruiz-Moyano et al. 2013). *B. breve* strains as mentioned, can however function as a ‘scavenger’ species in the infant gut with extracellular sialidases being the main source of cross-feeding interactions between *Bifidobacterium* strains. An example of this, is how *B. breve* UCC2003 successfully utilises sialic acid which is released by the sialidase activity of *B. longum* PRL2010 grown on 3’-SL (Egan et al. 2014b).

Other forms of mutualism are involved in the growth of *B. breve* strains in the gut of breast-fed infants (Sela and Mills 2010). This is likely due to the accumulation of LNB which can be consumed by other infant associated *Bifidobacterium* strains (Asakuma et al. 2011). LNB is known to selectively stimulate growth of four *Bifidobacterium* species most commonly associated with the infant gut, namely *B. bifidum, B. infantis, B. longum* and *B. breve*. LNB assimilation requires the presence and activity of an ABC transporter (GNB/LNB transporter) and a cytoplasmic phosphorylase (GNB/LNB phosphorylase) both of which are required for LNB metabolism. These proteins in the majority of cases are produced by the infant-associated strains of *B. bifidum, B. infantis, B. longum* and *B. breve* only (Xiao et al. 2010). The conservation of the GNB/LNB pathway in the four *Bifidobacterium* species most commonly associated with the infant gut (LoCascio et al. 2010) as well as the predominance of type I HMOs, suggest that it is quite likely that bifidobacteria have co-evolved along with their human hosts (Sela et al. 2008).

#### *Bifidobacterium catenulatum* subsp. *kashiwanohense*

*Bifidobacterium kashiwanohense* has been isolated from the stool of both healthy and anaemic infants, and is known to partially metabolise HMOs (James et al. 2019). While it is considered to be an infant gut-associated species, *B. kashiwanohense* is much less frequently identified when compared to the strains mentioned above with only a limited number characterised (Morita et al. 2015, Vazquez-Gutierrez et al. 2015). To date, little is understood about the ability of *B. kashiwanohense* to utilise HMOs, however, it has been shown to internalise and intracellularly hydrolyse low molecular weight, fucosylated HMOs, via the action of GHs, some of which are discussed below (Bunesova et al. 2016). This mechanism of utilisation of fucosylated HMOs is similar to that observed in *B. infantis*. It has also been suggested that *B. kashiwanohense* may grow on HMOs via cross-feeding activities of other HMO-utilising *Bifidobacterium* species present in the gut (Bunesova et al. 2016).

*Bifidobacterium kashiwanohense* APCKJ1, identified from the stool of a breast-fed infant, has the ability to consume fucosyllactose (James et al. 2019). This strain utilises fucosidase-encoding genes *fum*A1 and *fum*A2 along with transporter-encoding genes, *fum*S, *fum*T1 and *fum*T2 in the internalisation and hydrolysis of 2’-FL and 3-FL. It has also been shown that growth of *B. kashiwanohense* on both 2’-FL and 3-FL, results in an accumulation of lactate and acetate in the growth medium (James et al. 2019), two end products of Lac and fucosyllactose metabolism in bifidobacteria (Underwood et al. 2014). *B. kashiwanohense* APCKJ1 was grown on 1% 2’-FL and 3-FL resulting in production of the organic acid 1,2- propanediol (1,2-PD) a suggested end-product of L-fucose metabolism (Matsuki et al. 2016, James et al. 2019). Furthermore, key genes involved in L-fucose consumption as well as a putative pathway for the utilisation of L-fucose in *B. kashiwanohense* APCKJ1 when grown on 2’-FL have recently been described which are involved in converting L-fucose to L-2-keto-3-deoxyfuconate (James et al. 2019).

The presence of the FL transporter-1 and/or FL transporter-2 in bifidobacteria is an important factor in the ability of bifidobacteria to assimilate fucosylated HMOs while the SBP homolog gene of FL transporter-2 has been positively associated with the abundance of bifidobacteria in the gut of breast-fed infants (Garrido et al. 2016). Homologs of these FL transporters found in *B. longum* and *B. breve* have been identified in strains of *B. kashiwanohense* (James et al. 2019). The internalisation of fucosyllactose by *B. kashiwanohense* as well as hydrolysis by two α-fucosidase enzymes from the GH29 and GH95 families have also been characterised (Bunesova et al. 2016, James et al. 2019). The presence of genes encoding these fucosidases in certain strains correlates with the observed ability of such strains to consume fucosylated oligosaccharides, and while this ability has only been identified in the *B. kashiwanohense* strains with publicly available genomes, it is possible that this ability is a common feature of HMO metabolism in *B. kashiwanohense* (Morita et al. 2015, Vazquez-Gutierrez et al. 2015). To date, the ability of *B. kashiwanohense* to utilise LNT, LNnT, 3’-SL or 6’-SL to any significant degree has not been reported for the small number of *B. kashiwanohense* genomes characterised. *B. kashiwanohense* instead preferentially consumes 2’-FL and 3-FL from pooled HMO (Bunesova et al. 2016). The presence of a gene cluster BBKW_1838–1840 in *B. kashiwanohense* JCM 15439 has been identified and allows this strain to consume both LDFT and LNFP I in addition to the previously recognised 2’-FL and 3-FL (Sakanaka et al. 2019b).

The adaptation and ability to readily consume HMOs is an important factor in the establishment of *B. breve, B. longum, B. infantis* and *B. longum* in the breast-fed infant gut microbiome. While *B. kashiwanohense* is not yet recognised as a common infant-associated species, the presence of gene clusters containing *lac* and *fum* loci representing a possible HMO island which is explicitly dedicated to the metabolism of fucosylated HMO, provides an advantage for *B. kashiwanohense* establishment in the breast-fed infant gut. This, as well as its reported ability to utilise certain HMOs such as 2’-FL and 3-FL and both LDFT and LNFP I does certainly point towards an adaption to the infant gut environment (Sakanaka et al. 2019b). However, *B. kashiwanohense* does not seem to metabolise many of the other common HMOs found in breast milk such as LNT, LNnT, 3’-SL and 6’-SL (Bunesova et al. 2016). This perhaps explains its difficulty in becoming a dominant species when compared to *B. breve, B. bifidum, B. infantis* and *B. longum* in the breast-fed infant gut microbiome.

#### Bifidobacterium pseudocatenulatum

Genome sequencing of *B. pseudocatenulatum*, a frequently identified infant gut associated species which has various claimed health benefits including the ability to bind mutagenic aromatic amines and reduce cholesterol levels, has revealed the presence of specific genomic clusters which are involved in HMO utilisation. The ability of *B. pseudocatenulatum* to utilise LNB has been documented by Xiao *et al*. with 33 of 61 strains examined capable of growth on this substrate (Xiao et al. 2010). *B. pseudocatenulatum* LH11 identified from the faeces of a healthy breast-fed infant has been shown to grow in the presence of the core HMO structure LNT (Lawson et al. 2020). This study also identified the presence of genes encoding fucosidases from the GH95 family which are involved in fucosylated HMO metabolism in a number of additional strains identified from infant faeces. Most recently the ability of *B. pseudocatenulatum* to grow on 2% pooled HMOs as the sole carbohydrate source was examined with a number of *B. pseudocatenulatum* strains of infant origin shown to grow well on HMOs with many of the strains tested showing a consistent pattern of LNT and LNnT utilisation (Shani et al. 2022). The results of the study also indicated that fucosylated HMO utilisation was dependent on the presence or absence of genes encoding specific α-fucosidases from the GH29 and GH95 families. *B. pseudocatenulatum* strains SC585, MP80, MP86, JCM 7040, and DSM 20438 have also been shown to utilise an array of fucosylated purified HMOs including 2’-FL, 3-FL and LDFT, with strain MP80 also utilising LNFP I/III. Fucosylated HMO utilisation by these strains is facilitated by a gene cluster which encodes an ABC-type transporter and two α-fucosidases from the GH95 family. The highest growth levels on fucosylated HMOs were observed for strain MP80 which uniquely contains a second GH enzyme from the GH29 family as well as a Fuc mutarotase homologous to those observed in *B. infantis* ATCC 15697 (Shani et al. 2022).

### HMO metabolism by *Lactobacillus* species

While the majority of information available on the utilisation of HMOs is in relation to *Bifidobacterium* species, it has recently been shown that certain *Lactobacillus* species can metabolise particular HMOs to support their growth (Thongaram et al. 2017, Salli et al. 2021). The majority of information on HMO metabolism by lactobacilli is limited to strains of the phylogenetically related *Lacticaseibacillus casei*-*paracasei-rhamnosus* group (Rodríguez-Díaz et al. 2011). Three α-L-fucosidase enzymes involved in the metabolism of fucosylated HMOs have been characterised in lactobacilli, however, these are restricted to a select number of species. Sialidase enzymes involved in the metabolism of sialylated HMOs have not yet been described for this species (Rodríguez-Díaz et al. 2011). The α-L-fucosidases identified in *L. casei* have been shown to act on short, fucosylated oligosaccharides and the lack of an N-terminal signal sequence in their deduced primary structure suggests these enzymes are intracellular, perhaps taking up oligosaccharides and hydrolysing them inside the cell in a manner similar to that observed for many *Bifidobacterium* species (Rodríguez-Díaz et al. 2012). It has also been suggested that lactobacilli may utilise a ‘scavenger’ strategy similar to *B. breve* and *B. longum* whereby they may utilise short host-derived glycans released by the hydrolytic activities of other members of the gut microbiota (Zúñiga et al. 2018). The following sections will discuss studies which have investigated HMO metabolism in *Lactobacillus* species.

#### Lacticaseibacillus casei

*Lacticaseibacillus casei*, formerly *Lactobacillus casei* (*L. casei*) is a species associated with infant stool and probiotic supplements with strain specific immunomodulatory, anti-proliferative and pro-apoptotic properties (Abedin-Do et al. 2015). While knowledge on HMO metabolism capabilities of this species is limited, it has been reported that certain strains can, though to varying degrees, ferment specific HMOs to support their growth. Genome sequence analysis of *L. casei* BL23 has shown the presence of three genes encoding putative α-L-fucosidases of the GH29 family. These were identified as LCABL_20 390, which was previously annotated as α-L-fucosidase A (*alf*A), LCABL_28 270 (*alf*B) and LCABL_29 340 (*alf*C) (Rodríguez-Díaz et al. 2011) all of which have been shown to act preferentially on short fucosylated oligosaccharides. The low level of observed sequence homology between these enzymes (21% identity), suggests they have varying substrate specificities. AlfA releases α-1,6-linked Fuc residues from certain oligosaccharides while AlfB and AlfC have high activity on α-1,3 and α-1,6 bonds in fucosyl-GlcNAc, respectively (Rodríguez-Díaz et al. 2011). The excretion of Fuc into the supernatant as well as the lack of an N-terminal signal sequence suggest that these α-L-fucosidases are intracellular enzymes which perhaps take up fucosylated oligosaccharides and hydrolyse the structures inside the cell, similar to the α-L-fucosidases found in many *Bifidobacterium* species (Rodríguez-Díaz et al. 2012). These findings indicate that *L. casei* strains may utilise a scavenger strategy similar to that of *B. breve* in order to utilise small-mass host-derived glycans which are possibly released by the hydrolytic activities of other members of the gut microbiota (Zúñiga et al. 2018).

Growth of *L. casei* BL23 on GNB, LNB and LNT was examined using MRS media supplemented with these carbohydrate sources (4 mM concentration). This strain was shown to ferment GNB, LNB, and *N*-acetylgalactosamine in order to support its growth (reaching OD6_00nm_ values of between 0.6–1.0) but not LNT (Bidart et al. 2014). The phospho-β-galactosidase GnbG, of BL23 from the GH35 family was found to hydrolyse GNB and LNB, and to release Gal from LNT and also to hydrolyse Gal β1–6GlcNAc. Both GNB and LNB were transported and hydrolysed by the phosphotransferase system PTS^Gnb^ prior to hydrolysation by the phospho-β-galactosidase GnbG into *N*-acetylhexosamine and Gal-6P (Bidart et al. 2014). Growth of *L. casei* BL23 in the presence of lacto-*N*-triose (LNTri) as the sole carbon source has also been examined by monitoring its growth in MRS basal medium supplemented with LNTri as well as 3’-*N*-acetylglucosaminyl-mannose and 3’-*N*-acetylgalactosaminyl-galactose as the sole carbohydrate sources. This strain was found to grow well on 2 mM LNTri generating GlcNAc and Lac (Bidart et al. 2016). Upstream of the gene cluster *gnbREFGBCDA*, two genes, *bnaG* and *manA* are located. The *bnaG* (LCABL_02 870) gene belongs to the GH20 family and shows high specificity for β-1,3-glycosidic linkages especially *N*-acetylhexosaminyl β-1,3-linked sugars. BnaG is thought to be an extracellular enzyme due to the presence of an N-terminal signal peptide for secretion as well as a predicted C-terminal sortase-dependent cell-wall anchoring domain (Muñoz-Provencio et al. 2012). This enzyme hydrolyses LNTri into GlcNAc and Lac from outside the cell followed by transportation into the cells by a PTS or a PTS-independent permease (Bidart et al. 2016).

*Lacticaseibacillus casei* BL23 also metabolises the type II HMO component LacNAc for growth with the well characterised *lac* operon (*lacTEGF*) known to play a role in this metabolism. Utilisation of LacNAc is dependent on *lacE* and *lacF*, which encode the lactose-specific PTS^Lac^ enzyme II component CB (EIICB) and enzyme II component A (EIIA) domains, respectively, and *lacG* which encodes a phospho-β-galactosidase necessary for the hydrolysis of the intracellular phosphorylated Lac and LacNAc (Bidart et al. 2018). The ability of *L. casei* Lc-11 and *L. casei* DSM 20011 to sustain growth on 1% difucosyllactose (DFL), and Fuc was also examined with both strains found to be incapable of utilising either DFL or Fuc (Salli et al. 2021). *Lacticaseibacillus casei* Lc-11 has recently been shown by Salli *et al*. to support a minor level of growth on the fucosylated oligosaccharide 3-FL (Salli et al. 2021), The presence of α-L-fucosidases as well as catabolic pathways for the metabolism of GNB, LNB, LNT II and LacNAc and more recently an observed ability to utilise 3-FL indicate that *L. casei* can utilise a variety of HMOs in breastmilk (Zúñiga et al. 2020).

#### Lacticaseibacillus rhamnosus

Reports on HMO metabolism by *Lacticaseibacillus rhamnosus*, formerly *Lactobacillus rhamnosus* (*L. rhamnosus)* are rather limited with just a few documented studies. Whole genome sequencing of *L. rhamnosus* ATCC 53103 (*L. rhamnosus* GG) has revealed the presence of genes predicted to encode α-L-fucosidases (AlfA, AlfB and AlfC from the GH29 family) (Morita et al. 2009, Rodríguez-Díaz et al. 2011). Genome sequencing of *L. rhamnosus* HN001 also indicated the presence of α-L-fucosidase-encoding genes (Rodríguez-Díaz et al. 2012). The presence of the latter is rare in *L. rhamnosus*, and in lactobacilli in general, with *L. casei* the only other *Lactobacillus* species to date known to encode α-L-fucosidases which are capable of hydrolysing fucosylated HMOs *in vitro* (Rodríguez-Díaz et al. 2011). Many *L. rhamnosus* strains have been reported to utilise Fuc including *L. rhamnosus* strains GG and HN001 both of which grow well on 1% Fuc as the sole carbohydrate source and both of which encode genes involved in this Fuc metabolism that are well characterised (Thongaram et al. 2017, Salli et al. 2021). This feature however is strain-specific and not conserved across the species with other *L. rhamnosus* strains shown to be incapable of utilising Fuc for growth (Salli et al. 2021).

The *bnaG* gene is not conserved in the phylogenetically related *L. rhamnosus* species unlike *L. casei* and *L. paracasei* which partly accounts for the inability of most *L. rhamnosus* strains to metabolise the HMO LNTri. However, one particular strain of *L. rhamnosus*, HN001 despite the absence of the *bnaG* gene, has been shown to utilise the trisaccharide LNTri II as a carbon source for growth. This illustrates that despite the lack of the *bnaG* gene, certain *L. rhamnosus* strains can grow on LNTri II suggesting that in addition to the *bnaG* gene, there are additional enzymes present in lactobacilli which are important in the metabolism of this HMO (Bidart et al. 2016). Additional studies have also examined the ability of a number of *L. rhamnosus* strains to utilise HMO structures with *L. rhamnosus* GG found to be unable to grow on media supplemented with 1% 2’-FL, 3-FL or DFL as the sole carbohydrate sources. Three other *L. rhamnosus* strains examined in this study (Lrha_TS (DSM 20021), Lhra_HN001 and Lrha Lr-32) displayed rather modest growth on 1% 3-FL (Salli et al. 2021). The authors suggested that this inter strain variation among *L. rhamnosus* to utilise HMOs may be due to differences in Lac utilisation capabilities (Salli et al. 2021).

#### Lactiplantibacillus plantarum

*Lactiplantibacillus plantarum* formerly *Lactobacillus plantarum* (*L. plantarum*) is a commonly used probiotic species which inhabits the GI tract and is often isolated from infant stool (Lebeer et al. 2008). *L. plantarum* strains have been shown to utilise various carbohydrate sources for growth including fructooligosaccharides (FOS) and galactooligosaccharides (GOS), and the utilisation of HMOs and HMO-derived carbohydrate components has been suggested. While *L. plantarum* does possess genes that are predicted to encode enzymes capable of HMO hydrolysis such as GH2 and GH42 β-galactosidases, growth on HMOs however, is not often observed. Certain strains of *L. plantarum* such as LP-66 have been shown to successfully utilise 1% GlcNAc as a carbohydrate source (Thongaram et al. 2017, Zúñiga et al. 2018). This strain efficiently utilises 85% of this structure after 24 hours incubation (Schwab and Gänzle 2011). *L. plantarum* strain LP-66 exhibits limited growth on LNnT as the sole carbohydrate source (reaching OD600 reaching OD_600nm_ values of between 0.25 and 0.6), but can utilise free sialic acid for growth reaching OD values of between 1.0 and 1.4 (Schwab and Gänzle 2011, Thongaram et al. 2017). Indeed, *L. plantarum* strains are known to possess certain genes which are capable of free sialic acid metabolism (Almagro-Moreno and Boyd 2009). So-called *nan* clusters are gene clusters which are involved in sialic acid catabolism and such clusters have previously been described in *L. plantarum* strains (Zúñiga et al. 2018).

#### Lacticaseibacillus paracasei

*Lacticaseibacillus paracasei* formerly *Lactobacillus paracasei* (*L. paracasei*) is also a species often used as a probiotic and commonly isolated from the infant gut environment. Its carbohydrate metabolic capabilities are varied and inter strain differences are commonly observed as is the case for most *Lactobacillus* species. To date however, little research has been focused on the ability of *L. paracasei* strains to utilise HMOs (Watson et al. 2013). Thongaram *et al*. have recently shown that *L. paracasei* LCV-1 can utilise GlcNAc to support growth as the sole carbohydrate source (Thongaram et al. 2017). *Lacticaseibacillus paracasei* LCV-1 was cultured in MRS media containing 1% Lac, Fuc, GlcNAc, 2’-FL, 3-FL, 3’-SL, 6’-SL or LNnT. Growth levels were assessed by measuring OD_600nm_ values, which revealed that *L. paracasei* LCV-1 grew well on GlcNAc as the sole carbohydrate source present reaching OD_600nm_ values of between 1.4 and 1.8. This strain demonstrated no capacity for utilisation of the other HMO structures present such as 2’-FL, 3-FL, 3’-SL, 6’-SL, and LNnT when tested at the same concentration (Thongaram et al. 2017). *Lacticaseibacillus paracasei* Lpc-37 has recently been shown to be capable of moderate growth on the fucosylated HMO 3-FL, but there are no further reports confirming this ability to date (Salli et al. 2021).

#### Lactobacillus acidophilus

*Lactobacillus acidophilus* strains are known to possess genes encoding enzymes involved in hydrolysing certain HMOs, yet seem to generally incapable of growth on HMOs with growth being very strain-dependent (Schwab and Gänzle 2011). One such strain which exhibits moderate growth on particular HMOs is *L. acidophilus* NCFM (Marcobal et al. 2010). Genome analysis of *L. acidophilus* NCFM confirmed the presence of a large repertoire of genes which are predicted to be involved in carbohydrate utilisation such as 20 PTS and five ABC transporters. These transporters often share a genetic locus with glycosidases and transcriptional regulators, allowing for localised transcriptional control. While the exact roles of these genes remain largely unknown for NCFM, it is possible that some of these genes are involved in HMO metabolism (Altermann et al. 2005). Specifically, *L. acidophilus* NCFM has been shown to utilise the neutral, type II HMO LNnT, with growth of between 0.6 and 1.0 measured using OD_600nm_ on 1% LNnT as the sole carbohydrate source present (Thongaram et al. 2017). The utilisation of LNnT involves an extracellular β-galactosidase (*lac*L, LBA1467) which cleaves the terminal Gal molecule of LNnT for growth, while leaving the resulting trisaccharide LNTri II in the medium undigested. This ability to utilise LNnT is dependent on *lacL* activity, with inactivation of *lacL* resulting in abolished growth of *L. acidophilus* NCFM on LNnT (Thongaram et al. 2017). While *L. acidophilus* NCFM was shown to be capable of utilising LNnT, this strain is incapable of metabolising other HMOs such as 2’-FL, 3-FL, 3’-SL and 6’-SL. The ability to utilise LNnT has also been observed for *L. acidophilus* LA-5 which grows on LNnT as a sole carbohydrate source, but does not appear to utilise other HMOs (Thongaram et al. 2017).

*Lactobacillus acidophilus* NCFM, *L. acidophilus* LA-5 and *L. acidophilus* LA-14 are known to encode β-galactosidases belonging to the GH2 and GH42 families (Schwab et al. 2010). The presence and activity of β-galactosidases from GH2 and GH42 as discussed earlier, (Marcobal et al. 2010) imply that *L. acidophilus* may also be capable of metabolising certain HMOs (Goh Klaenhammer 2015). *Lactobacillus acidophilus* NRRL B-4495 is capable of utilising purified HMOs pooled from human milk in order to support growth with Wang *et al*. examining the ability of *L. acidophilus* to utilise specific carbohydrate sources such as Glc, HMOs, GOS, xylooligosaccharide (XOS) and 2’-FL for growth (Wang et al. 2016). Following 48 hour incubation with each of these carbohydrates as the sole carbohydrate source at 2%, *L. acidophilus* NRRL B-4495 was found to consume HMOs from pooled HMO (26 ± 2%) and also moderately consume the individual HMO 2’-FL (35 ± 3%) which was additionally tested as the sole carbohydrate source present. From the pooled HMOs, NRRL B-4495 was found to consume HMOs LDFT (29 ± 3%), LNT (19 ± 2%), LNFP (18 ± 2%) and LNDFH (21 ± 4%) to varying degrees (Wang et al. 2016). HMO fermentation by *L. acidophilus* strains results in the production of organic acids such as lactic acid and butyric acid with trace amounts of propionic and valeric acid also present (Wang et al. 2016). The ability of *L. acidophilus* to utilise HMOs or HMO-derived monosaccharides has also been described for *L. acidophilus* FUA3191 which has been shown to successfully utilise HMOs including 2’-FL, 3-FL, LNT, and 6’-SL as measured by HPEAC-PAD (Schwab and Gänzle 2011).

## Potential of next generation probiotics to utilise HMOs

Traditionally, probiotic strains have been isolated from the human gut and fermented foods. As the function and commercial use of bifidobacteria and lactobacilli have become well established, newly identified gut commensals with purported health benefits, such as *Akkermansia muciniphila, Bacteroides* spp., *Faecalibacterium prausnitzii, Ruminococcus bromii*, and *Roseburia* species, have emerged. Notably, the abundance of many of these bacteria is inversely correlated to several disease states (Zhou and Zhi 2016, Xu et al. 2021, Bahena-Román et al. 2022). These next generation probiotics have been identified using new tools such as next generation sequencing techniques and bioinformatics, which has aided in the isolation of these commensal bacterial species given their commercialization potential. Recent research has begun to explore methods to selectively enhance their growth *in situ* with a limited number of studies (described below) investigating their potential to utilise HMOs.

### Akkermansia muciniphila

*Akkermansia muciniphila* (*A. muciniphila*) is a Gram-negative, mucin-degrading anaerobe from the phylum *Verrucomicrobia* which represents between 3% and 5% of the gut microbiota in healthy individuals (Belzer and de Vos 2012). Administration of *A. muciniphila* has been shown to demonstrate benefits comparable with current probiotics and studies have shown that *A. muciniphila* is associated with several health benefits including reduction of the risk of obesity and type II diabetes and improved intestinal barrier integrity (Derrien et al. 2004, Cheng and Xie 2021). Further to this, in adults a decreased abundance of *A. muciniphila* in the gut microbiota is associated with metabolic dysfunctions (Dao et al. 2016), hypertension, ulcerative colitis and inflammatory bowel disease (Rajilić-Stojanović et al. 2013). Meanwhile, in infants reduced *A. muciniphila* colonization has been shown to negatively correlate with a compromised immune system and atopic dermatitis (Lee et al. 2018). For these reasons *A. muciniphila* has been considered as a promising candidate next generation probiotic (Cheng and Xie 2021). While mucin is the preferred growth substrate for this species, it has recently been shown that a limited subset of *Akkermansia* strains may utilise HMOs for growth (Kostopoulos et al. 2020). One strain of *Akkermansia muciniphila*, Muc^T^ has been found to grow on human milk and utilise HMOs by employing a selection of glycoside hydrolase enzymes including those from GH2, GH20, GH29, GH33, GH35 and GH95 families (Kostopoulos et al. 2020). Incubation of *A. muciniphila* in human milk resulted in the production of considerable amounts of acetate, propionate and succinate and the release of Glc and Gal as a result of utilisation of Lac and HMOs from breast milk. HPLC analysis was used to investigate the utilisation individual structures including 2’-FL, 3-FL, DFL, LNT, LNFP, 6’-SL and 3’-SL. HPLC profiles demonstrated that *A. muciniphila* consumed > 95% of 2’-FL and 3’-SL present when incubated in 10% human milk (Kostopoulos et al. 2020). Following this, the ability of *A. muciniphila* to grow on 10% either 2’-FL or 3’-SL as the sole carbohydrate source was investigated with both carbohydrate sources supporting growth (as measured using OD_600nm_). Growth on 2’-FL also resulted in the production of 1,2-PD, a suggested end product of Fuc metabolism as mentioned above.

In a recent study by Luna *et al*. growth of *Akkermansia* strains on five individual HMO structures (2’-FL, 3-FL, LNnT, 6’-SL, and LNT) was examined using a basal medium supplemented with the HMOs in a background of mucin, mimicking the carbon sources present in the infant gut environment (Luna et al. 2022). One strain in particular, CSUN-19 exhibited considerable growth on the HMOs tested versus the media containing no HMOs as measured by OD_600nm_. In order to confirm utilisation, HMO concentrations were measured before and after 48 hours incubation revealing that strain CSUN-19 utilised 64% of HMOs present, while *A. muciniphila* Muc^T^ utilised > 93% of available HMOs (Luna et al. 2022). HMO utilisation by *Akkermansia* is thought to be mediated by the use of extracellular GHs present which cleave HMOs into their constituent monosaccharide or disaccharide components (Luna et al. 2022). It has been suggested that the close structural similarities between mucin and HMOs may explain the ability of these bacteria to utilise both glycan types as *Akkermansia* possess an array of GHs for the effective metabolism of mucin including α-fucosidases, β-galactosidases and β-acetylhexosaminidases (Kostopoulos et al. 2020). Indeed, the presence of *A. muciniphila* in the infant gut may be in part due to its array of glycan-degrading machinery including enzymes involved in mucin and HMO degradation, which aid in the metabolism of a broad range of HMOs and their constituents (Kostopoulos et al. 2020).

### Bacteroides

*Bacteroides* are common constituents of the gut microbiota and are known for their ability to utilise a range of oligosaccharides, polysaccharides and host-derived glycans, especially mucin (Marcobal and Sonnenburg 2012). A number of *Bacteroides* species such as *Bacteroides thetaiotaomicron* have been suggested as potential next generation probiotics (Tan et al. 2019) due to their potential for positive health outcomes such as reducing obesity and insulin resistance as well as reduction of intestinal inflammation (Sears et al. 2008, Zuo et al. 2011). Members of the genus *Bacteroides* have been shown to use milk glycans such as HMOs as fermentable carbohydrate sources for growth (Marcobal et al. 2010). When grown on HMOs, expression of specific gene clusters referred to as polysaccharide utilisation loci (PULs) which are involved in mucin consumption are induced. It may again be that due to the structural similarities between mucins and HMOs, *Bacteroides* can degrade HMOs using parallel metabolic strategies. *Bacteroides* species first bind complex oligosaccharides on the cell surface before hydrolysing which enables transit through the outer membrane into the periplasm followed by their degradation. (Marcobal et al. 2011).

*Bacteroides thetaiotaomicron* has been shown to utilise HMOs, growing efficiently on minimal medium (MRS containing no glucose) supplemented with 1.5% pooled HMOs as the sole carbohydrate source and consumes a broad range of HMO structures (Marcobal et al. 2011). The ability of *Bacteroides* to utilise HMOs was also recently examined with *Bacteroides dorei* and *Bacteroides vulgatus* shown to grow on purified HMOs including 2’-FL, DFL, 3’-SL, 6’-SL, LNT and LNnT as sole carbohydrate sources. As is the case for most HMO utilising species in the infant gut, strain-to-strain variation was evident across multiple strains of the same species (Kijner et al. 2022). *B. dorei* underwent an extensive metabolic response to the presence of HMOs, upregulating many shared GHs but not those which are directly known to degrade HMOs. *B. dorei* utilised the HMOs tested for growth as the sole carbohydrate source present reaching an OD_600nm_ of 0.6–1 after 24 hours growth on 2’-FL, DFL, 3’-SL, 6’-SL, LNT, or LNnT (Kijner et al. 2022). *B. fragilis* (Bfra_TS), *B. vulgatus* (Bvul_TS) and *B. thetaiotaomicron* (Bthe_TS) type strains have also been shown to grow well on 1% 2’-FL, 3-FL, and DFL as the sole carbohydrates present (Salli et al. 2021).

### Roseburia

Clostridiales from the *Roseburia-Eubacterium* group are typically associated with the adult human gut microbiome, yet are also a core member of the infant gut microbiome (Vital et al. 2017). The abundance of these bacteria is often decreased in patients with metabolic, inflammatory and cardiovascular diseases with its benefits often associated with the production of butyrate which exerts many immuno-modulatory effects as well as reducing the risk of cancer, atherosclerosis and enteric colitis (Bultman 2018). For these reasons, *Roseburia* species have emerged as potential next generation probiotics. *Roseburia hominis* and *Roseburia inulinivorans* have been observed to grow on HMOs from breast milk. Growth analysis on purified individual HMOs revealed the ability of *R. hominis* to grow on LNT, LNB, and GNB, while *R. inulinivorans* utilised LNB and GNB as well as sialic acid (Pichler et al. 2020).

## Concluding remarks and future perspectives

It is important to review the current understanding of the growth abilities of particular gut bacteria and individual bacterial species on HMO for a number of reasons. Highlighting the species and strain level interactions that could support probiotic development is essential especially for non-breast-fed infants of which high rates of infection has been associated when compared to HMO consuming breast-fed infants. The individual species discussed here represent some of the most widely used probiotics on the market. The health benefits of probiotics are often strain-specific, and the use of each probiotic strain should include accurate identification of the strain through genetic and phenotypic analyses. The functionality of probiotics should be disclosed *in vitro* and *in vivo* so that they can be used as legal functional ingredients. Examining the ability of the collective infant gut microbiome to utilise HMO still requires technological and bioinformatic advancements although some studies are emerging that are beginning to address this need (Chleilat et al. 2020). With more HMOs becoming available, it is possible to supplement formulas with blends of HMOs to formula-fed infants again emphasising the importance of understanding the growth abilities of individual species to utilise individual HMO.

Apart from particular species of the genera *Bifidobacterium, Lactobacillus, Bacteroides* and *Akkermansia*, relatively few other gut bacteria are known to metabolise HMOs. Members of the gut microbiota which catabolise HMOs do so using an arsenal of specific glycoside hydrolases such as α-fucosidases, α-sialidases, β-galactosidases, β-acetylhexosaminidases and lacto-*N*-biosidases. The HMO utilisation capacity of infant-associated species has been most extensively studied in *Bifidobacterium*, particularly in high HMO utilising species such as *B. infantis* and *B. bifidum* as well as *B. longum* and *B. breve*. In comparison, there are considerable gaps in our knowledge regarding HMO utilisation by other species. Recent advances in genome sequencing technology have substantially aided in the identification of putative glycan transporters and glycoside hydrolases which are generally associated with HMO metabolism. However, further research is required to determine the exact functions of these transporters and enzymes in many commensal bacteria. It is possible that many bacterial species thrive on the by-products and metabolites which are released by HMO metabolism by certain commensals present namely *Bifidobacterium*. This potential ‘cross-feeding’ has numerous benefits such as generating positive metabolic co-dependencies and interactions between members of the infant gut microbiota leading to a network which forms the basis of the mature gut microbiota with important positive health implications for later life. To fully understand the complex process of HMO utilisation strategies employed by infant associated bacterial species, it is perhaps the use of multi ‘omics’ technologies such as genomics, metabolomics, proteomics and meta-transcriptomics which may prove the most efficient in the future.

Combining the health-promoting properties of HMO with live microorganisms can result in next generation synbiotics or ‘HMObiotics’. Similar to the probiotic and prebiotic fields, the future of such synbiotics will be influenced by the development of novel strains and the manufacture of preferred HMO substrates, informed by and targeted to specific microbiome niches in the gastro-intestinal tract of individuals. Such combinations have potential for supplementation in many products aimed at individuals at different life stages or those suffering or susceptible to various disease states where lower numbers of health-promoting bacteria such as bifidobacteria are evident. Examples include infant milk formula or as food supplements for infants and toddlers with the aim of improving the discrepancy of *Bifidobacterium* cell numbers found between breast-fed and formula-fed infants. Such compositions may also have potential in products aimed at benefiting the elderly, immunocompromised individuals, individuals on antibiotics or indeed as a means for treating or preventing diseases associated with lower numbers of commensal bacteria such as inflammatory bowel diseases (Crohn's disease, irritable bowel syndrome, ulcerative colitis), periodontal disease, rheumatoid arthritis, atherosclerosis, allergy, multi-organ failure, asthma, and allergic diseases (Slingerland et al. 2017).
